# Practical Application of a Relationship-Based Model to Engagement for Gene-Drive Vector Control Programs

**DOI:** 10.4269/ajtmh.23-0862

**Published:** 2024-06-18

**Authors:** Ana Kormos, Lodney Nazaré, Adionilde Aguiar dos Santos, Gregory C. Lanzaro

**Affiliations:** ^1^ Vector Genetics Laboratory, University of California, Davis, California;; ^2^ University of California Malaria Initiative, University of California, Davis, California;; ^3^ Ministry of Health, Delegacia de Saúde Distrital de Água Grande, São Tomé and Príncipe

## Abstract

Engagement is an important component in the advancement of gene-drive vector control research programs as developers look to transition the technology from the laboratory to the field. As research advances and engagement surrounding this novel technology is put into practice, knowledge can be gained from practical experiences and applications in the field. A relationship-based model (RBM) provides a framework for end-user development of engagement programs and strategies. The model places end users at the center of the engagement decision-making processes rather than as recipients of predetermined strategies, methods, and definitions. Successful RBM application for healthcare delivery has previously been demonstrated, and the University of California Malaria Initiative (UCMI) has applied this model to its gene-drive program in the Democratic Republic of São Tomé and Príncipe. The model emphasizes the importance of local leadership in the planning, development, and implementation of all phases of project engagement. The primary aim of this paper is to translate the model from paper to practice and provide a transparent description, using practical examples, of the UCMI program implementation of RBM at its field site. End-user development of the UCMI engagement program provides a unique approach to the development of ethical, transparent, and effective engagement strategies for malaria control programs. This paper may also serve as a reference and example for projects looking to establish an engagement program model that integrates end-user groups in the decision-making processes surrounding engagement.

## INTRODUCTION

The University of California Malaria Initiative (UCMI) is a not-for-profit research collaborative working in partnership with government and stakeholder groups in the Democratic Republic of São Tomé and Príncipe (STP). The UCMI mission is to contribute to malaria eradication through genetic modification of populations of the African mosquito vector, *Anopheles coluzzii,* rendering them incapable of transmitting malaria parasites to humans.^1^ The UCMI population modification strategy is designed to eliminate the malaria parasite without eliminating the mosquito.

### The UCMI program objective and field site selection.

Our aim is to conduct an ecologically confined field trial of a genetically engineered mosquito with a low-threshold gene drive. We have selected a field site that maximizes prospects for success, minimizes risk, and serves as a fair, valid, and convincing test of efficacy and the impacts of a gene-drive product intended for large-scale deployment in Africa.^2^^.^

The UCMI program believes that island sites are the ideal environment for achieving this aim. Applying the WHO guidelines and definition for an ecologically confined field trial,^3^ the UCMI conducted a rigorous evaluation of potential island field sites, which concluded that the island nation of STP would be an optimal site.^2^

The nation of São Tomé and Príncipe consists of two volcanic islands located 140 km apart in the Gulf of Guinea, roughly 250 and 225 km, respectively, off the northwest coast of Gabon. The larger island, São Tomé, has a total area of 854 km^2^ and a human population of about 193,000, and Príncipe has a total area of 142 km^2^ and a population of 7,324 people.^4^ It is a Portuguese-speaking country with a young population (about half of the population is under 18 years of age).^5^ Its remote location and small size and population limit the development and economic activities in the country, resulting in elevated poverty rates (15.6%), lack of employment opportunities, and government reliance on external financing.^5^

Human occupation of the STP is recent, having been initiated roughly 500 years ago with the first wave of Portuguese colonization.^6^ Malaria and its mosquito vector (*A. coluzzii*) were introduced into STP at about the same time.^7^^,^^8^ Malaria is now endemic in the STP, and although it was considered to be in the pre-elimination phase in 2009, the current trend indicates otherwise.^9^ The number of malaria cases in the STP have been increasing each year since 2020, a nearly 30% increase in malaria cases from 2020 to 2021^10^ and a nearly 50% increase from 2021 to 2022.^11^ The National Malaria Control Program in the STP reported a similar increase in the number of cases in 2023, and in the first 12 weeks of 2024, cases have been nearly 50% higher than they were in the same reporting period in 2023 (unpublished data provided by the National Malaria Control Program).

In late 2018, the UCMI program presented the government of STP with an evaluation of island sites and information about program technology and objectives. Discussions regarding a partnership with the Ministry of Health and the National Malaria Control Program began, and with this, a relationship was initiated between the UCMI and STP. The discussions surrounding partnership and collaboration included the subjects of field research and baseline data collection, capacity strengthening and training, and engagement.

### Engagement for gene-drive research.

Advancements in the development of gene-drive technology for malaria control have accelerated the need for effective engagement practices and have stimulated discussion and debate surrounding best practices, recommendations, and guidelines for meeting this need. Gene-drive technology has the potential to spread and persist in nature, presenting important questions related to its scope and impact over space and time. This also means that the scale of human populations potentially affected may be quite large and, without opportunity to “opt out” of the intervention or participate in decision-making about its use, raises different sets of ethical and engagement questions.^12^ In addition, public perceptions and attitudes about science and technology are dynamic, and they influence expectations and opinions about how the public ought to be engaged and involved. Although no industry standard yet exists to evaluate gene drive,^13^ numerous publications have offered conceptual frameworks and guidance for gene-drive developers in consideration of these questions.^3^^,^^12^^–^^32^ In many of these publications, “co-development,” shared decision-making, and participatory development are articulated as important, if not essential, elements in the development and application of the technology. The recently published second edition^3^ of the WHO Guidance for Testing Genetically Modified Mosquitoes places importance on “a co-development approach that emphasizes authentic partnership and knowledge engagement” for community engagement and development of the technology in general. Other papers analyzing engagement practices and the obligations implicit in them have focused specifically on community consent for the use of the technology.^33^^–^^35^ Inherent in these concepts is the call for developers to reflect on their empowered positions coming into a field site and the need to develop engagement programs that acknowledge this and move to shift power back to the end-user groups.

### The UCMI approach to engagement.

We agree about the importance of establishing an approach to engagement that is inclusive—shaped by existing knowledge, partnerships, and collaborations. We also agree in the importance of end-user involvement in decision-making surrounding engagement. We define *end users* as the population who may be recipients of the technology, which, for the UCMI program, are the residents of the STP. End users include stakeholders and community members alike. We define *communities* as all who are impacted by project work, equaling all residents of the STP, and *stakeholders* as group(s) of individuals who are not only impacted by the project work but who may also be involved in governance surrounding the project and may also participate in defining the level of community acceptance required for this project. Decision-making and the level of involvement of one versus another is determined again by the existing governance and social structures established within the end-user population.

The UCMI program leaders established value-based beliefs and objectives in the initial stage of research that shaped the program’s approach to engagement. Included in the program mission is the stated importance of working in partnership and collaboration with stakeholders and community members in an ethical and transparent manner and to establish open dialogue and relationships of trust with our partners and collaborators. We believe that engagement frameworks and strategies should be developed on a case-by-case basis, reflective and inclusive of the perspectives, values, and knowledge of the local population where the technology is being developed.^26^ We also believe that questions and definitions concerning community acceptance and consent should be answered by the end-user groups where the technology is being considered, rather than predetermined by external experts and academics not directly engaged in the work, and that a shift in power and decision-making is essential.^28^ Establishment of a national engagement program developed by end users for end users provides a means for answering these important questions and a foundational starting point for assessment.

### The UCMI model for engagement.

The UCMI has selected a model for engagement that supports end users in leading the development and implementation of the engagement program, strategy, and communication from start to finish. The goal of UCMI engagement is to ensure program transparency, share knowledge, and provide end users with what they need to make an informed opinion about whether to support the use of the technology or not. Fully integrating stakeholders and engagement practitioners from the STP in the development of all aspects of the engagement program reduces opportunities for preconceived and/or predetermined strategies developed by the research program or other external groups of experts from being imposed on end-user groups. It also increases opportunities for regular community member participation, building on existing relationships and pathways for communication and knowledge sharing.

Prior to selecting a field site or initiating engagement, the UCMI program consulted with an engagement practitioner with experience implementing the relationship-based model (RBM) for public health interventions. The model is one that has been tried and tested.^36^^–^^43^ It is a model initially developed for transformation of healthcare delivery, articulating the importance of relationships between the providers and the recipients of care and the importance of involving the recipient in the decisions surrounding the delivery of care.^43^ The concepts of the model are further advanced in The Nuka System of Care developed by the Southcentral Foundation in Alaska, shifting decision-making power entirely to the end users of the healthcare system, transforming care delivery to meet Native Alaskan values and needs.^36^ The Nuka System of Care recognized that if a public health intervention is to be successful, it should be developed by the end user, reflective of their values and voices. This is particularly important when end-user groups historically have been recipients of inequitable power dynamics and decision-making. Southcentral Foundation offers a “core concepts” training focused on the importance of an RBM in engagement with people and commnuities.^44^ Core concepts and guidelines for applying the model taught at the Southcentral training have been adapted and applied by the UCMI.

The model emphasizes the importance of end-user involvement in answering the how, who, what, where, and when questions surrounding the development of an engagement program. It recognizes the importance of relationships in the integration of existing knowledge, resources, strengths, and beliefs in decision-making.

The decision made by the UCMI to adapt the RBM to its program evolved from the program’s commitment to the establishment of trusting relationships with our field site partners and the need for integration of existing knowledge, practices, and perspectives into decisions surrounding engagement at the field site.

Establishing relationships through knowledge sharing between program and end-user groups to inform program practice, principles, and target goals and establish a balance of power was a primary aim of the program. Development of trust as a foundation of a relationship requires a lot of time, effort, patience, and resilience from everyone involved. This is a core concept in the RBM and the foundation on which the UCMI engagement strategy in the STP was developed.

### Application of the RBM for gene drive.

The RBM offers a framework for engagement that acknowledges the critical importance of human knowledge and participation in the success and acceptance of public health interventions. The RBM can be applied at an individual level or a community or population level. The type of application and intervention plays a role in determining who should be involved and the size and scope of the engagement effort. In many cases, the RBM has been used for interventions that an individual could choose (or not) to use. In other cases, it has been applied in health delivery models to adjust how end users receive and access care,^35^ in which case, not every individual may agree on the final decision or choose to give an opinion. The application is determined on a case-by-case situation. The importance of the application is that is provides all end users an opportunity to be involved in engagement and have access to knowledge and information that inform their choices, opinions, and decisions.

The question then remains, how do we scale this model up to engage with an entire nation and apply it to an intervention for malaria control that may not present each individual citizen with a yes or no choice about whether to use it or not? This is the question we attempted to answer here. The UCMI has taken the core concepts of the RBM and applied them to the development and implementation of a national engagement program for gene drive. This has been done in a phased approach, with each phase dedicated to establishing relationships of trust, knowledge sharing, and end-user involvement.

## MATERIALS AND METHODS

The UCMI has previously articulated its commitment to the RBM and the approach that was taken to initiate stakeholder engagement in the STP.^25^ We outline below the methods the UCMI used in applying the RBM in the initial development and implementation of the engagement program in the STP from 2019 to 2023.

### Initial stakeholder engagement: Establishing trust through shared understanding and goals.

The UCMI program established an initial collaborative relationship with the Ministry of Health (MoH) in the STP in 2019. This is the ministry responsible for developing the national malaria control strategy and the governance of external partners who wish to participate in the malaria control effort.

Inherent in the core concepts of the RBM is the acknowledgment of established systems and values. These include existing laws, policy, national priorities and processes, regulation and governance, and national beliefs (both historical and current) within the country. This complements the WHO’s guidance for the evaluation of genetically engineered mosquitoes, which states the importance of adapting to current governance rather than replacing it.^3^ It is this initial relationship, which established program understanding of the local governance, history, societal and political structures, and priorities, that directed all future UCMI engagement actions.

The program relationship with the MoH guided establishment of other important program relationships with stakeholders actively involved in the national malaria control and elimination effort. These stakeholders included the National Malaria Control Program, nongovernmental organizations, associations, the National Public Health System, the national university, government agencies, and representatives from international organizations (WHO and the United Nations Development Program) representing diverse interests and areas of expertise. Participation expanded further on the recommendations of the members of this group to include others from civil society and community leaders. This interdisciplinary group of individuals guided the development of the UCMI engagement program and will be referenced in this paper as the *engagement stakeholder group* (Table 1). This group further explored existing social values and priorities, expanding the program’s understanding of priority issues, and the common societal behaviors and responses reflective of shared values in the STP.

**Table 1:** Description of roles and responsibilities of the UCMI engagement program team in the STP

Position/Role	Description	Functions and Responsibilities
Engagement Stakeholder Group	Interdisciplinary group including the Health Delegation, National Malaria Control Program, and public health agencies: Members of this group were recommended by the Minister of Health.	Provided advice and consultation to the UCMI team and Ministry of Health about engagement and communications for the UCMI program; developed communication plan, engagement strategy, and engagement team structure
Health Delegates	Medical doctors (seven) assigned to one of seven health districts in the country: Each delegate was responsible for all health care delivery including community health awareness and education in their district; health delegates were appointed by the Minister of Health.	Provided direct supervision of all engagement work in their district; selected community health agents for UCMI project, supervised the preparation of the monthly engagement activity report, conducted monthly team meetings, participated in district- and national-level awareness activities, participated in monthly meetings with UCMI engagement manager, and provided consultation regarding engagement plans and activities
Engagement Manager	Dedicated full-time UCMI position that managed all aspects of engagement in the STP; STP national with experience and training in communication and engagement, experience working in public health, and extensive experience in the STP conducting engagement	Acted as a direct liaison between UCMI program administrators, field science team, and engagement team, and developed and operationalized engagement plans; provided direct support and training for engagement team members, collaborated with local graphic designers and engagement stakeholders in the development of materials and social media content
Malaria Focal Points	Trained malaria technicians (six) assigned to one of six health districts of São Tomé: They reported directly to and were assigned by the health delegate in their district.	Provided support and coaching for community health agents; participated in UCMI engagement and awareness activities in their district; completed monthly activity report for UCMI engagement activities; assisted in the development of engagement plans for their district and participated in all UCMI program trainings and team meetings
Engagement Focal Point–Príncipe	Dedicated full-time UCMI position that supervised and led all engagement activities in the Autonomous Region of Príncipe; STP national who lived in Príncipe and had experience conducting engagement and health education in Príncipe. This position reported to the engagement manager.	Provided direct support and supervision for the Príncipe engagement team, which included teachers and community health agents; provided monthly activity report to engagement manager, led, and participated in engagement and awareness activities with stakeholders in schools and within the communities in Príncipe
Teachers	Licensed biology teachers who were STP nationals and worked in the primary and secondary school system: Teachers reported to the Ministry of Education, and those who worked with the UCMI engagement team were selected for the project by the Ministry.	Led engagement activities in the schools; conducted science fairs and educational scientific demonstrations in the street and at health fairs; assisted in scientific trainings for community health agents; directly supported engagement activities in the communities; conducted engagement activities for faculty and staff in schools
Community Health Agents	Community health educators who conducted health education and awareness within the health district where they lived: They report to the health delegate in their district.	Conducted awareness and educational activities in their health district; attended UCMI-provided trainings and educational workshops; attended UCMI engagement team meetings; assisted in the development of engagement plans for their district

STP = Democratic Republic of São Tomé and Príncipe; UCMI = University of California Malaria Initiative.

Establishing relationships with a large and diverse group of stakeholders takes time. This is an important reason why engagement should begin early, well in advance of initiating fieldwork. The UCMI program team conducted numerous information-sharing workshops, presentations, one-on-one meetings, virtual meetings, E-mails, and phone calls with stakeholders for nearly 2 years (between 2019 and 2021). This early engagement applied skills for healthy dialogue and communication, such as the use of open-ended questions (e.g., “How should we develop an engagement program?”) and active listening and reflections (e.g., “We are understanding that stakeholders want a national engagement effort; is this accurate?” followed up with open-ended question[s]: “How can we achieve this, and who should be involved?”). Several important questions (Table 2) were the focus of group discussions. Reports and written documentation of meetings and action items further reinforced important messages and information shared with the UCMI team and gave stakeholders an opportunity to further refine and clarify directions and perspectives until a decision point was reached (Table 2). These practices are used consistently by the UCMI team in our interactions with end-user groups.

**Table 2:** Description of key decision-making points in the development of the UCMI engagement program in the STP, including the decision-making path, end point decisions, and the involvement of end users in the decisions

Initial Work/Pre-Conditions Creation of Internal Field Site Dossier – Literature Review and Field Site Scoping Trip Established Points for Contact with National Malaria Control Program in STP during Initial Scoping Visit				
Decision Point/Question(s)	Decision-Making Path	Decision-Making Authority	Decision End Point	Stakeholder Involvement in Decision
Who is the decision-making authority in the STP that the UCMI should contact for permission to conduct research and establish a partnership agreement?	The UCMI program contacted National Malaria Control Program, which referred the program to the Minister of Health. The UCMI met with the Minister of Health. The Minister of Health suggested stakeholder workshops in the STP. The UCMI co-hosted stakeholder workshops with the Ministry of Health.	Ministry of Health	Minister of Health established a collaborative partnership agreement with UCMI to conduct field and laboratory research, provide training and capacity building, and conduct engagement.	STP stakeholders: Ministry of Environment, WHO, United Nations Development Program (UNDP), National Malaria Control Program, health delegacy, University of the STP, Ministry of Agriculture, national NGOs, private advisors to the Minister of Health, and the national media participated in the stakeholder workshops and provided input and opinions regarding the UCMI program, objectives, and scope of the partnership agreement.
How should the UCMI program establish an engagement program in the STP?	Dialogue was initiated in the stakeholder workshops and in meetings with Minister of Health. Stakeholders suggested additional workshop with STP malaria control partners and managers of existing engagement efforts. Workshop was conducted with engagement stakeholder group and UCMI. The UCMI presented RBM model and commitment to local partners leading engagement. Met with health delegacy and mapping of STP health system and existing public health engagement.	Ministry of Health	UCMI engagement program will be national, use the existing engagement structure within the health districts, and work in direct collaboration with the health delegacy.	Dialogue among national stakeholders identified engagement strategies and practitioners in the STP and their scope of work, training, funding, and areas of expertise. From these discussions came a recommendation for integration of the UCMI engagement program with the health delegacy and its teams of community health agents who conducted public health engagement and awareness activities for local and international health initiatives.
What should the UCMI engagement strategy look like?	The UCMI asked health delegates this open-ended question in a meeting to discuss engagement team structure. Health delegates suggested developing a proposal for the Ministry of Health. Health delegate from largest district put together a draft document outline with a phased plan for engagement. The document was reviewed by all delegates, UCMI team, and engagement stakeholder group. Final document was shared with Ministry for approval.	Ministry of Health Health Delegates	A general plan for the engagement strategy was described, outlining an iterative and phased approach to engagement for both stakeholder and community groups, building on existing knowledge and relationships and further defined by the engagement team and the communities where these teams worked.	Health delegates and the stakeholder engagement group developed the document with technical input from the UCMI. The general strategy was based on their own knowledge and experience working with stakeholders and community members in the STP. The Ministry of Health reviewed the document, provided suggested edits, and approved the final draft.
How is the UCMI engagement team structured? What are the roles and responsibilities?	UCMI met. with the health delegacy and engagement stakeholder group to build UCMI team capacity and understanding surrounding current engagement in STP, including roles, responsibilities, budget, knowledge, and existing resources. Community engagement practitioners met with UCMI to share perspectives about community knowledge and perceptions related to malaria, existing challenges, language requirements, engagement methods and materials, and differences among communities. UCMI met with health delegates to discuss initial funding availability and areas where the program could lend support for training and capacity building. UCMI met with UNDP administrators and Ministry of Health representatives to discuss national compensation rates and financial expectations and requirements to support engagement work in STP. Health delegates met with UCMI to discuss strategy, team composition, roles, and responsibilities, training requirements, and team selection process. UCMI team and engagement manager met with Ministry of Education about involvement of teachers	Ministry of Health, Ministry of Education, Health Delegates	Ministry of Health approved a UNDP-developed budget for engagement activities using the model that the health delegates proposed. A two-phased approach to the UCMI engagement team was established. The team was distributed among all seven health districts in the country and was developed based on available funding, population size within each district, and community distribution within each district. Phase one was developed considering the initial UCMI engagement budget, and phase two was developed to request additional funds to support advancement of engagement in the STP. Term of reference was established for all roles, and the recruitment process and training needs were identified.	Engagement practitioners participated in open dialogue with health delegates and the UCMI team. They contributed important information about how engagement was being conducted, what was working and what was not, and what kind of language and materials should be used in different districts/communities. This information was used by the health delegates in the development of the term of reference and team size and distribution within the districts. Health delegates directly determined the size and distribution of the engagement team, term of reference, and recruitment process of team members. The Ministry of Health assigned specific teachers to the engagement team based on term of reference, previous experience, and areas of expertise. The UNDP and Ministry of Health contributed national compensation rates, per diem, and travel rates for engagement partitioners, and they shared financial expectations and requirements needed to support capacity building and engagement activities based on similar international partnerships and national government standards. (The UNDP has historically been an important partner to the Ministry of Health in management of malaria projects and other health-related projects).
How do we develop the initial engagement activities?	Recruitment and hiring of engagement manager and identification of district-specific community health agents (CHAs) were conducted after a process outlined by the health delegation and engagement stakeholder group. Health delegation met with engagement manager and UCMI team; UCMI asked this open-ended question, which initiated group discussion. Engagement manager and engagement team workshops and trainings were conducted that included discussions regarding experiences with district and community-specific engagement activities in the past (what worked/challenges/community preferences). Suggested first step was to conduct assessment in all districts to guide development of activities. It was also suggested that activities be developed by district-level teams, acknowledging that communities and populations across the country were different and had different priorities and preferences.	Engagement Team	A National community assessment was conducted to determine district-specific gaps in knowledge about malaria, perspectives about malaria, and preferences regarding information sharing and engagement. Assessment results were used by each district-level engagement team to determine a first-phase engagement activity plan and activities.	Engagement team members worked together to arrive at decision end points. Decision process, end point, and subsequent decisions about how to conduct the assessment, where to conduct it, and its development were all determined by the engagement team.
How do we develop initial training for the engagement team, and who should be involved?	Health delegation made initial suggestions. Suggestions were shared with engagement team members, and team members provided input about their own preferences. The UCMI, health delegation, engagement manager, and engagement stakeholder group discussed potential STP collaborators with expertise in subject areas. Stakeholders contacted STP professionals and invited them to participate. Health delegates contracted STP public health professionals who had previous experience facilitating communication and group exercises. The UCMI provided content and material related to the project and the technology. The engagement manager met with the health delegacy to finalize agenda and practical components for the training.	Engagement Team, Collaborators/ Experts in STP	Training was conducted over 4 days with a focus on general UCMI program information, objectives, and activities, malaria biology, malaria control in the STP, malaria detection and treatment, and review of general communication and engagement skills with practical components. STP collaborators who were experts in these areas were involved in developing the content and material for training and provided presentations.	The health delegation, engagement manager, and engagement stakeholder group worked together to identify collaborators, agenda development of practical group activities and application of skills, and evaluation tools for the training. Collaborators (e.g., national malaria control entomologists, malaria focal point, and RN responsible for malaria treatment, medical doctor, NGO technician responsible for IRS, etc.) developed presentations and materials for training components and questions and activities for evaluation.
With whom are we engaging?	General engagement strategy identified “communities,” all of which were impacted by project work, equaling all residents of the STP and “stakeholders” as group(s) of individuals who are not only impacted by the project work but who are involved in the project and will define the level of community acceptance required for this project and provide oversight and direction of project. Discussions and engagement mapping of stakeholders were led by engagement manager, expanded on early STP stakeholder list provided by Ministry of Health. Collaborators and partners including CHAs and health delegacy asked for input and suggestions. Stakeholder list and engagement groups determined in engagement team meetings.	Engagement Team	The engagement plan included three specific groups, with a specific plan for each group: 1. Stakeholders 2. Schools (primary to university) 3. Communities.	Full participation and decision-making of the engagement team in the final decisions and planning surrounding who is engaged with, and the who, how, what, and when questions within the plan for each specific group.
How do we engage with communities?	Engagement team meetings were conducted to discuss this question. The CHAs, health delegacy, and engagement manager shared their historical knowledge and experience conducting engagement in their communities in the STP. The CHAs discussed with community members and leaders in their districts how they wanted to be engaged. Information and ideas from communities within each district were shared with the larger team. Engagement activities were planned with this information.	Engagement Team, Communities	Engagement in communities is ongoing, and the plan included monthly activities and participatory meetings. Each district was different, and some of the activities included community-based meetings led by community leaders, individual visits at community members’ homes, meetings at health centers, participatory radio programs, social media, health fairs, and pop-up demonstrations in community centers. The UCMI project has an office in a central location where community members can drop in and speak to project team members, share information, and express concerns/questions.	Community members and leaders discussed and shared with the CHAs who lived and worked in their communities/district. This information was taken back to the district team and the larger national team. Plans and activities were determined by the engagement team and integrated the input and preferences shared with the CHAs who worked and lived in these communities. These activities and plans were tailored to each district by the district-level engagement teams. Plans and activities were continuously adjusted and improved based on this input.
How do we engage with stakeholder groups?	Engagement team meetings were conducted to discuss this question. The engagement manager conducted meetings with engagement stakeholder group to discuss this question. STP stakeholders were identified from earlier mapping exercises engaged. Stakeholder groups requested specific methods of information sharing and engagement. The engagement team developed a plan for engagement using requests from each group.	Engagement Team, Stakeholder Groups	Engagement with stakeholders is ongoing and has included different activities for each group depending on preferences. Activities included information sharing meetings and workshops, scientific training and capacity sharing activities, public consultation events for open dialogue and questions and answers, monthly activity planning meetings and review of project data, protocols, and engagement plans. Meetings were held to develop engagement material content, bi-monthly newsletters, publications in national news, videos and interviews on national TV and radio, social media, E-mails, and quarterly project progress reports.	Stakeholder groups in the STP participated in discussions with the engagement team to share preferences and opinions about how they wanted to engage with and be engaged by the project. This information was integrated into the stakeholder engagement plan. Regular feedback and dialogue with stakeholders drove continuous adjustment and improvement of stakeholder engagement activities.
How do we engage with the schools?	The UCMI team and engagement manager had initial meetings with Ministry of Education leaders. Ministry of Education presented a proposal for specific teachers to integrate into the engagement team. Teachers met with the engagement team. Teachers developed a proposed plan for engagement in schools. Teachers and the engagement manager met with school administrators to refine the engagement plan in schools.	Engagement Team, Teachers, Ministry of Education	School engagement activities were conducted during the regular school year in the STP and included a variety of activities depending on the school and educational level of students. Activities included individual classroom demonstrations, participatory learning exercises with scientific models, malaria activity booklets and other visual aids, school assemblies, informational booths and tables, malaria-related coursework and classes, educational contests, and take-home activities.	The school engagement plan was developed by teachers and school administrators who had knowledge and expertise working in the schools in the STP. Regular feedback and input from these groups drove continuous adjustment and improvement of activities that occurred in the schools. Student assessments and participatory activities have been conducted to integrate student perspectives and feedback into the decision-making surrounding future activities and materials used for school engagement activities.
How do we define community acceptance of the UCMI technology?	The UCMI asked this question of STP stakeholders, specifically government leaders. The government made clear that it will make this decision. Path to making this decision will be determined by STP leaders and is under consideration.	STP Stakeholders, Government Leaders	Final decision end point to be determined.	Stakeholders in the STP who will make decisions about the UCMI technology will determine the answer to this question. Stakeholders have requested that the UCMI project continue to support ongoing engagement activities.
What is the objective of UCMI engagement?	The UCMI developed early objectives within the program plan and funding proposal. The UCMI team and engagement stakeholder group had initial discussions to refine and adjust the UCMI objectives to fit the STP goals. Early objectives determined with stakeholder group were later refined with the engagement team members.	UCMI Team, Stakeholder Group, Engagement Team	This was a national engagement effort with the intent of reaching all members of society using a phased approach to ensure that informed decisions about the technology (acceptance and use of) could be made and to ensure transparency at every stage of the project. Opportunities for information sharing and dialogue were available to all.	The stakeholder engagement group refined the original objective of UCMI engagement to specifically include all members of society, to clearly define a phased approach and the phases, and the importance of creating opportunities for all members of society to engage and share information.
How will the success of engagement be measured or evaluated?	The engagement stakeholder group developed integration of annual assessments to evaluate engagement. The engagement team developed assessments and tools (online surveys/games) to evaluate activities, messages, materials, and understanding at each phase. The engagement team developed a timeline for assessments and target goals; goals were refined based on assessment results and community feedback. External social science team engaged to conduct national assessment at the end of year 3 to evaluate overall knowledge, perception, and acceptance	Stakeholder Group, Engagement Team	Assessments (annual) and online surveys (quarterly) were to be conducted nationally to adjust engagement strategies and respond to community knowledge, understanding, perceptions, and concerns. Measures of success were determined by question type/category (e.g., knowledge of malaria, project work, and objectives – 80%). Success goals are to be measured at the end of 2024: access to engagement, overall understanding of UCMI objectives and goals, understanding of technology, perception and acceptance of technology.	The engagement stakeholder group initially suggested assessments to evaluate knowledge, understanding, and perceptions of engagement for each phase. The engagement team further refined assessments, timeline for conducting assessments, and the target goals for each assessment. The engagement team refined activities and strategies based on results. Assessment results determine whether adjustments to target goals need to be made and when and how to integrate new information.

CHA = community health agent; IRS = indoor residual spraying; NGO = nongovernmental organization; RN = registered nurse; RBM = relationship-based model; STP = Democratic Republic of São Tomé and Príncipe; UCMI = University of California Malaria Initiative; UNDP = United Nations Development Program.

Inclusion of end users in program development is an important RBM concept and is critical in establishing an engagement program that understands and respects the values and perspectives of communities and that builds on existing strengths and resources. It is essential in the development of project messaging and communication and in the mitigation of project misunderstanding and misinformation. The UCMI, under the direction of the MoH, collaborated with the engagement stakeholder group to 1) develop a phased communication plan and engagement strategy, 2) identify a local engagement team structure, and 3) develop terms of reference for engagement team members. This work was the result of the first phase of stakeholder engagement. Through establishment of shared understanding and goals, this important end-user group guided the early development of the UCMI engagement program.

### Identification of a local engagement team: Building on existing strengths and resources.

The STP engagement stakeholder group identified a team that initially included national community health agents (CHAs). These CHAs provide community-based education and awareness activities for national health programs and work under the direction of the health delegation in the communities where they live. Most of the CHAs had previously conducted awareness programs about malaria in their communities, had received basic training in malaria education, and had a deep understanding of community composition, language, and values. Their inclusion in the UCMI engagement team was important for these reasons but, most importantly, because they had established relationships of trust and respect with community members and with their colleagues in the health system. They knew what to do and how to do it. The RBM tells us that as local health educators, as well as potential end users of the technology, these are the individuals who should be involved in engagement decision-making.

Also included in the UCMI engagement team are the health district malaria focal points and health delegates. The STP contains seven defined health districts, each of which has its own health delegate (medical doctor) who coordinates and manages the health activities and care delivery in that district, including the work of the CHAs. Health delegates are responsible for coordinating malaria testing, treatment, and communication in their district, and they work closely with the National Malaria Control Program in data reporting and surveillance. The malaria focal points liaise with the health district and the malaria control program to coordinate delivery of malaria control activities and education. They are often trained malaria control technicians, and they report directly to the health delegate in their district.

Several information-sharing workshops and planning meetings were organized with the health delegates and malaria focal points in 2021 to discuss project objectives, engagement budget, engagement program structure, and a general communication plan. The inclusion of these groups in the engagement program was critically important from the perspective of the RBM because it gave decision-making authority to those who are in the best position to make informed decisions. Over a period of 6 months, this group further developed and refined a phased engagement strategy and demonstrated that they had established relationships with the CHAs and communities in their districts, a deep understanding of the national health priorities and challenges, and a commitment to the success and safety of the members of the engagement team.

The engagement strategy included broad timelines and objectives for engagement consistent with larger UCMI program objectives and goals. These included a national engagement program; phased information sharing guided by assessments of knowledge, perception, and understanding; direct collaboration with the national health systems; and activities, messages, and materials directed and developed by a local engagement team. Phases of information sharing were identified as small building blocks, each building on the next set of concepts so that understanding of more challenging aspects of the technology (e.g., gene drive and population modification) could be achieved slowly over time. Initial phases of information were defined as general malaria facts, malaria transmission, malaria control in the STP, general UCMI program information and field research (e.g., mosquito collections, mosquito population studies), malaria transmission and parasite lifecycle, control methods and the impact on the mosquito and parasite, UCMI-modified mosquito, beneficial genes and how they interrupt the parasite life cycle, gene drive and how it works to modify the population, and how the modified mosquito works as a malaria control tool. These phases were established with the understanding that assessments of community understanding, perception, and knowledge would inform the focus of each phase and information would be developed to address the community feedback provided. In addition, health delegates and malaria focal points worked to revise the initial UCMI engagement budget, establish terms of reference for the CHAs supporting the UCMI engagement program, and determine a process for CHA selection. Discussions surrounding CHA time commitments to other health projects and available resources informed this work. These early discussions and group work evolved into what is now an engagement leadership team that has taken responsibility for management and assessment of CHA project work, leading capacity building and training activities, and the regular evaluation and planning of engagement activities.

The term of reference for a local STP manager for the engagement program was also defined. The UCMI team collaborated with a smaller group of engagement stakeholders to recruit and select candidates for this position. The selected candidate met all the criteria defined in the terms of reference, most importantly, 5+ years of engagement experience in STP communities; an advanced understanding of STP political and social structures, values, beliefs and culture; and exceptional communication skills.

The addition of a dedicated, full-time engagement manager in the fall of 2021 was a turning point in the establishment of the engagement team and strategy. The manager collaborated with the health delegates to identify a total of 55 CHAs for the UCMI engagement program. Available funding and estimated days of work/month determined this number. Estimations of CHA time were derived largely based on their previous experiences working with Global Fund malaria control projects that included a community education component associated with a particular intervention. There was historical knowledge about time required to conduct door-to-door engagement and community workshops in each community. District size and population determined the CHA number in each district.

Although the initial UCMI program budget included a robust engagement component, it was created using estimates and approximations. It was understood by the project funder that the project would require a second phase of funding for ongoing activities at the field site after establishment of agreements and partnerships, which was a consideration in the early development of the team. While revising the original budget, they also developed a projected phase two budget. This included additional funding for training, capacity strengthening, and increased time and for engagement activities as the phases of the engagement increased in complexity.

On review of the initial communication plan, which included engagement in schools, the manager recommended that the program partner with the Ministry of Education to integrate biology teachers into the program for successful engagement with students. A term of reference was developed, and in collaboration with the Ministry of Education, five biology teachers were initially selected to work with the UCMI engagement team. At the end of 2021, a full engagement team was established (Table 1). It is important to note that the roles and functions of the team members outlined in Table 1 were largely preexisting. Many of these positions (health delegates, teachers, malaria focal points, CHAs, and stakeholder group members) were not dedicated full-time to the project or direct employees of the UCMI. This is consistent with how the RBM works, integrating a new program with existing resources, values, and governance.

### Team-based approach: End-user development of engagement plans and activities.

The RBM took a team-based approach that recognized that every member of the UCMI team in the STP (field research, laboratory research, advisors, etc.) plays a role in engagement.^27^ Engagement activities are integrated and coordinated with the work of the local research teams. The engagement manager facilitates regular communication with the engagement stakeholder group, research teams, and other local advisors and consultants to ensure consistent communication and information sharing among all groups.

The RBM emphasizes relationships, shared understanding, and active participation in the development of community-based activities. This consideration and inclusion ensures that program activities meet community members where they are, leveraging their perspectives, knowledge, and expertise into the decisions involved in the development and implementation of the UCMI engagement program. This was the most important part of the initial work and is detailed in Table 2.

Decisions that shaped and determined the approach to engagement were made by local authorities and stakeholders through dialogue with one another and with community members. Decision-making processes were defined as the specific steps and actions taken to reach a decision end point. *Decision-making power* was defined as the group (or groups) who have authority to make end point decisions. Authority in many cases is determined by the existing structure of governance and hierarchy within the STP, and in some cases, it is determined by the terms or reference for specific leadership positions defined by these existing authorities or governing bodies.

Specific engagement activities, UCMI field research activities, and UCMI project planning are influenced by what is learned through engagement (Table 3). In the space of a publication, it is difficult to describe all of the project learnings from engagement with end-user groups; however, Table 3 is intended to illustrate some specific examples of how dialogue with the community shaped and defined UCMI project work in the STP.

**Table 3:** Examples of dialogue with end users and integration of their perspectives and preferences in UCMI project decisions

Engagement Activity	End-User Group Engaged	What Was Learned through Dialogue	Project Actions/Decisions
Initial National Community Assessment (2021)	Communities	In response to Question 13 regarding preferred sources of information, many community members shared their preference for TV/radio and went beyond this to tell the CHAs that social media communication and video access on mobile telephones are preferred and how they would like to access/share information.	The engagement manager started project’s YouTube and Facebook accounts. A local graphic designer and communications professional was contracted to work with the engagement team to develop videos and other visual aids. Photos and updates about project work and engagement activities are regularly posted on Facebook. The CHAs have discussed the availability of information and materials with community members and provided the Facebook link and UCMI website address. All content is available on mobile devices. Project phone and data plans are provided to all engagement team members so that the CHAs can share information in real time with community members.
Community-Level Engagement in Each District (Door to Door and Community Meetings) to Share Information about Initial UCMI Project Fieldwork Collecting Mosquito Larvae from Sites Across the Country	Community Leaders and Community Members	Some of the communities (and community leaders) requested more information through public consultations in their community center as a follow-up to one-on-one engagement, some community members were interested in participating in the collection process, and some community members specifically wanted to know if the UCMI could, and would, kill all the mosquito larvae.	The engagement and field teams scheduled public consultations/community meetings in partnership with community leaders where requested. Community members who expressed interest in participating in the collections were contacted by the field team when the work was being conducted in these districts, training was provided, and community members participated with the field team in the collections. The engagement team began giving more detailed information about the scope of the UCMI project and the research we had permission from the government to conduct (which did not include larval control or larvicide applications to kill mosquito larvae).
Community Engagement Activities (Door to Door and in Community Meetings) Aimed at Sharing Information with Communities about the Objective of the UCMI Program	Community Members	The CHAs reported four common comments and themes that they encountered across districts and communities: 1) “Why can’t they just kill all the mosquitoes?” 2) “What will happen if the mosquito bites me?” 3) “Will the UCMI mosquito kill the other mosquitoes?” and 4) “How long will the efficacy of the beneficial genes last in the mosquitoes?” Concerns about the project reported by the CHAs primarily centered around harm the bite of a modified mosquito might cause to people.	The UCMI discussed concerns about human harm from the bite of a modified mosquito with stakeholders and the engagement team. Several stakeholders and CHAs suggested conducting a biting demonstration using the modified mosquito to evaluate the difference in bite response between the modified mosquito and the wild type of mosquito in the STP. The UCMI team developed a demonstration protocol, solicited volunteers at the University of California Davis, hired a media and communications team, and conducted a biting demonstration as suggested by stakeholders and CHAs. The demonstration was filmed and translated into Portuguese. This video was posted on the project website and was shared with the engagement team, who then shared it with community members. In addition, the engagement team developed information and short videos to share with communities to address the other common themes/questions that were expressed. These videos were created and posted on Facebook and were shared with communities during engagement activities.
Project Information-Sharing Workshop with Science Students and Faculty at the University of the STP, Which Included Informal Presentations and Open Forum for Questions and Answers	University Students and University Faculty	University students were very interested in access to information about genetics, molecular biology, bioinformatics, and modeling. They asked UCMI if the team could provide guest lectures and seminars on these subjects. One of the biology professors requested a series of lectures for his biology students focusing on malaria and malaria biology. A few of the students requested an opportunity to work in the field collecting mosquitoes with the field team. Faculty asked about mentorship opportunities and advanced studies and training for biology students at the university.	The UCMI conducted a survey with biology students after the workshop to identify specific themes and subjects that they were interested in. The results of the survey were used to plan a series of guest lectures and seminars at the university in partnership with the faculty. These began several months after the first workshop and have continued with each new school year. The UCMI worked directly with the biology professor at the university to develop a series of in-person lectures focused on malaria and malaria biology. These were conducted within the same year. Students who expressed interest, in addition to a biology professor, joined the UCMI field team in the field and assisted in mosquito collections. The UCMI, in partnership with our collaborators at the Institute for Hygiene and Tropical Medicine (IHMT) in Portugal, has mentored three fourth-year biology students in the STP who were interested in conducting research in medical entomology. We are currently mentoring a fourth student. The UCMI secured funding to provide four students from the STP with advanced degree scholarships to study in Portugal at the IHMT. The project partnered with stakeholders and university faculty to develop a recruitment, evaluation, and interview process for student candidates.
Engagement in Communities to Share Information about Plan to Conduct UCMI Mark, Release, Recapture (MRR) Studies (Door to Door, Public Consultation, Community Meetings)	Community Members	Communities expressed concern that the mosquitoes being released for the MRR study were modified mosquitoes and had many questions about that step. Communities where the MRR studies were to be conducted also expressed a desire to be involved in the study. There was also interest from community members, as well as stakeholders, in receiving results of the study.	Prior to the study, the UCMI engagement team and graphic designer worked to produce an animated video to explain the steps and processes involved in the study. A second 30-second video was created to specifically address the misconception about the release of modified mosquitoes during the MRR study. Both videos were shared with communities and were shared broadly on national TV and radio. The CHAs conducted follow-up engagements in the communities where the studies were to be conducted to follow up after video release, and there was a greater understanding of the study and less concern about the mosquitoes that were to be released. The malaria technicians from the National Malaria Control Program worked with the UCMI engagement team to identify community members who were interested in participating in the study and who had availability to participate and to attend training. Between 25 and 30 volunteers from the communities participated in each MRR study. After results from the MRR studies were analyzed, the field science team and the engagement team worked together with the graphic designer to create an animated video summarizing the results of the study, and this was shared on national TV and social media.
Distribution of a UCMI Malaria Education and Activity Booklet in a Primary School; Booklets Distributed to Every Student; Teachers Partnered with Engagement Team to Provide Malaria Education, and School Administrators Conducted Student Assessment of the Booklet	Primary School Students, Teachers, School Administrators, and Parents	Students were engaged during interactive presentations with the engagement team members and asked a lot of questions about the UCMI project and malaria in the STP. Teachers expressed interest in conducting additional activities that provided education about malaria, and many students and parents shared interest in additional activities and education about malaria in the STP. Parents and teachers shared the booklet with others in their community and social circles. As a result, the engagement manager was contacted by parents and teachers from other schools requesting copies of the booklet. Several teachers and administrators from other schools requested the same engagement in their school with the booklet.	The engagement manager and the teachers in the engagement team conducted meetings with other school administrators and developed a plan for integration of the booklet into school curriculums in São Tomé in 2024. An engagement team will follow up with the primary school where the pilot was conducted to meet with the teachers and administration about planning follow-up activities with the students for the 2024 school year.
Engagement with Community Members about Malaria Transmission*	Community Members	Dengue appeared in the STP in early 2022. During community engagement activities focused on malaria transmission, community members expressed concerns about recent cases of dengue. There were many who were concerned that dengue was related to malaria. There was limited knowledge about dengue. One of the concerns included a perception that the UCMI-modified mosquito had been released and that the modified mosquito was transmitting dengue. There were many community questions about dengue prevention, transmission, and treatment because there was not any national public education or awareness happening to inform communities about the epidemic, and the request for more information was overwhelming.	The UCMI responded to this request by conducting informational meetings and public consultations with stakeholders and, more specifically, the Ministry of Health. The UCMI engagement team worked with stakeholders and the research teams to develop an illustrated fact sheet about dengue versus malaria, which was distributed nationally. This was the only educational information being distributed and was also promoted by the Ministry of Health on its social media sites. The UCMI engagement team developed a short video explaining the differences between the two diseases and the vectors, and this was shared on social media and national TV. The engagement team also conducted focused community engagement activities to address community concerns (that had been shared on social media, with CHAs, and with stakeholders) about dengue, the relationship of dengue to malaria and the UCMI-modified mosquito, and differences in the controls for the disease.
Distribution of 60-Second Animated Videos to Address Common Questions and Concerns from Community Members and Stakeholders	Community Members and Stakeholders	Engagement team members regularly recorded and reported to the engagement manager common questions and concerns received while conducting engagement activities or in general conversations. These were also recorded in the community assessment in 2022. At a group training after assessment, the team reviewed these concerns and grouped them based on common themes. Six common concerns (themes) emerged. It is the practice of the team to respond to community concerns directly in face-to-face dialogue or community meetings. Community feedback from the November 2022 assessment pointed to the importance of using social media in the delivery of information.	The UCMI engagement team worked together to identify common concerns and misconceptions that needed to be addressed. They identified six based on community and stakeholder feedback: 1) the UCMI has modified mosquitoes in the STP, 2) modified mosquitoes will come from the United States, 3) the modified mosquito brought dengue to the STP, 4) the project will release modified mosquitoes (no permission needed), 5) the bite of the modified mosquito will cause harm to humans, and 6) all mosquitoes in the STP will be modified. Together they worked with the local graphic designer, stakeholder engagement group, field team, and other stakeholders to develop short animated videos of characters in dialogue discussing the concerns and the facts surrounding each issue. These videos were then released on social media, national TV, and YouTube and shared in engagement activities in the communities.

CHA = community health agent; STP = Democratic Republic of São Tomé and Príncipe; UCMI = University of California Malaria Initiative.

* These activities coincidentally occurred during the appearance of dengue in the STP.

Early dialogue with community members was intended to establish a baseline understanding of community knowledge and perspectives related to malaria, the UCMI program, and preferred methods of engagement. The engagement team determined that the best way to answer the question “What is the most effective way to conduct engagement?” was to ask the community members (Table 2). The team developed a quantitative assessment designed to gather information from communities across the country.

### Community assessment 2021.

The initial assessment was developed with the engagement team, stakeholders, UCMI team, community leaders, and other public health professionals. The aim of the assessment was to gather baseline data about community knowledge, perspectives, and preferences related to malaria and malaria engagement in the STP. The assessment was conducted in November 2021 in 29 communities across all seven districts in the country. The number of communities included in each district was determined by the total population size in the district (larger number of communities for more populated districts), and specific communities within each district were randomly selected. Twenty individual assessments were completed in each community for a total of 580 responses. This number was determined by available resources (financial, human, and required transportation and supplies). Individuals were randomly selected from 20 different households, and an effort was made to assess an equal number of male and female participants. All individuals who participated in the assessment were over 18 years of age.

The results of the assessments were recorded, and the initial analysis was conducted by a University of STP student in collaboration with a University of California Davis administrative assistant and research assistant (Supplemental Table 1). Results were analyzed by the engagement manager, health delegates, and the UCMI team, and findings were shared and discussed with stakeholder groups involved in developing the assessment. The results demonstrated that each district had different perspectives on malaria and current malaria controls, but overall, nearly 80% of the population was concerned about malaria, 70% reported knowing little about malaria transmission, and preferred methods for receiving health information were through community meetings and digital media (TV/radio/Facebook).

An initial community engagement plan and district-specific activities were developed by the team to address the results of the 2021 assessment. Focused activities applied community-preferred methods of engagement and focused on education about malaria transmission. The CHAs shared the results of the assessment with members of the communities in January 2022 using visual summaries of the data specific to each district and community. The plan also included sharing general information about UCMI program objectives, team composition, and the initial field biology work and baseline data collection being conducted in partnership with the National Malaria Control Program. In addition, the CHAs identified a process for responding to community questions, concerns, and grievances to ensure that community feedback and opinions would be continuously integrated into the engagement planning processes.

Engagement plans for schools and stakeholder groups were developed using a similar process. Each plan tailored for the specific groups (e.g., primary schoolchildren, school faculty, community leaders, university students, government officials) was based on feedback and suggestions received during initial engagement activities with students and teachers in classroom presentations and with stakeholders during workshops and information-sharing events (Table 3).

Engagement plans for each group (communities, schools, stakeholders) included a written guide for the engagement team. These described goals and objectives during specific time frames that built one upon the other to ensure a shared understanding among all groups about the program research, goals, and objectives. The engagement manager played a critical role in coordinating collaboration and communication within the entire program team. The manager provided leadership and direction in the development and implementation of engagement activities coordinated within each stage or phase of program research. Bringing together the engagement stakeholders and the UCMI field biology research team ensured that engagement activities were fully integrated with the UCMI field biology research. The development process was realized through coordinated, integrated capacity strengthening to determine the most effective engagement activities to build understanding and acceptance. A detailed example is shown below using mark, release, recapture (MRR) studies conducted in the STP.

### Engagement for MRR studies.

The UCMI program previously identified specific, standard practice field biology studies that needed to be done to provide information to national stakeholders that was essential in their eventual evaluation of the UCMI technology. Mark, release, recapture studies within the natural population of mosquitoes was one of these. The MRR studies evaluated the dispersal and size of the mosquito population.

The development process began by sharing the established protocol with stakeholders who would be involved in implementation and study design, including scientists, public health leaders, malaria program leaders, scientific advisors, and local engagement and entomology teams. The protocol was revised based on their feedback, and a shared set of goals for the study were established. Revisions included adjustment to specific sites where MRR studies were to be conducted based on accessibility and allocation of resources, time periods for studies based on historical weather data and local knowledge of weather patterns, and training and identification processes of volunteers assisting in the study. The final protocol was then sent to the STP Health Ethics Committee for evaluation and approval. The work in creating a study design and protocol with a multidisciplinary stakeholder group was possible because of the relationships of trust that had been previously established with these groups. Group members understood the program and the objectives and trusted that their opinions would be valued and integrated into the study design.

After the shared set of goals, design, and protocol had been established, the engagement team collaborated with the local field team and community leaders to develop an engagement plan for the study, including key messages and information that would be essential to share with community members and a training program for the engagement team specific for the study. Months prior to the study, local field and engagement teams implemented the training for the engagement team and conducted public consultations in communities where MRRs would be conducted. Comments and concerns from the communities (Table 3) were then used to further refine the study plan and to inform the engagement team in its development of activities leading up to the study.

The local engagement team developed a specific plan that included numerous awareness activities specific for the communities in and around the study, as well as communities distanced from the study. The goal was to inform the entire country about the study and the planned outcomes. An animated video and narration were created explaining the study methods, objectives, and measured outcomes and shared on national TV and radio and social media. Poststudy data and results were also shared using an animated video and through face-to-face awareness activities across the country.

This example demonstrates how inclusion of end-user groups in decision-making encourages relationships of trust and ensures shared understanding and program transparency. Together, the engagement stakeholders, field research team, and engagement team developed engagement activities that fully integrated with field research activities while adapting to the specific concerns and feedback of the end-user groups being engaged.

### Developing an environment that supported the model.

Successful implementation of the RBM depended on bringing people together to share, collaborate, create, and participate in the program development. It also relied on establishing a system of gathering and responding in real time to feedback from the end-user groups being engaged. Establishment of an environment to support the work (encouraging good communication and providing safe spaces for open communication and sharing) is an essential component of the model. The UCMI program considered this as a foundational step in building the program and provided participatory communications training for staff, leaders, and researchers in the fundamental concepts of the RBM, as well as general communication skills and methods to support open dialogue, collaboration, and trust. The training was provided by a communications consultant with experience in training public health professionals and community groups to apply the RBM for program development projects.

The same training was developed for the local teams in the STP in partnership with the consultant and local STP public health professionals who had previous communications trainings and experience with health education programs. Provision of participatory communications training is an invaluable component for successful RBM implementation and has been a priority area of investment for the UCMI program. The engagement team has received quarterly, 3–5-day trainings over the last 18 months that included multiple interactive modules combining formal presentation, group work, practical exercises, and assessments of knowledge and understanding through pre- and posttests for each module. These trainings were codeveloped and delivered by local engagement stakeholders, malaria control stakeholders, educators, and social scientists. Every training included a communication and engagement module focused on supporting good communication and dialogue. Aims of the trainings included providing an environment for team building, information sharing, and capacity strengthening surrounding the complex research and science behind the project but, more importantly, the capacity to establish the same safe sharing environments in their communities where they conduct engagement activities. Training modules included UCMI program objectives and work, malaria biology, malaria transmission, control and treatment, engagement communication, RBM model, engagement methods and importance, communication skills with practical applications, responding to conflict and concerns, and development of engagement activities. To reinforce skills learned in training, the engagement team within each district met monthly and worked together to support good communication and information-sharing practices. Materials and messages for the monthly engagement activities in the communities were reviewed, and small group practice exercises were facilitated by the engagement manager within each district team.

Additional training and participatory team meetings and learning activities were conducted for members of the field and engagement teams and UCMI program leadership and administrators. Regular weekly meetings between the program engagement, field science, and administrative team leaders encouraged the active practice of open dialogue and sharing of information. This information was then transferred to the larger engagement team to help inform its development of program activity plans and materials. Monthly meetings with stakeholder groups provided an opportunity for the engagement manager to review materials (videos, booklets, posters, etc.) with a multidisciplinary team who provided advice and feedback. This ensured accuracy of content, and it promoted transparency and awareness about what information was being shared in the communities between all project stakeholder groups.

Continuous evaluation and quality improvement were important components of the UCMI program and the RBM, one that allowed the program to gather and respond to feedback and opinions of end-user groups, further expanding the opportunity for shared decision-making.^27^ Engagement activities and personnel were regularly evaluated, and information from these evaluations were used to inform future program planning. Annual community assessments were conducted to evaluate engagement activities and methods as well as community knowledge and perceptions. Any response indicating understanding of less than 75% required adjustment, any response indicating utilization of an engagement tool or activity with lower than 50% was adjusted, and any response indicating misperception or misunderstanding of greater than 25% required immediate action. Annual exams and skill evaluations were conducted for CHAs and teachers within the engagement team. This included a written exam (passing was 80% or higher) and an oral exam where each individual was asked to conduct an engagement activity for a panel of engagement stakeholders and knowledge and skills were evaluated (80% ranking was required). Community health agents, teachers, and malaria focal points evaluated program trainings (presenters, information, and activities) to inform how trainings could be improved. Engagement leaders were evaluated annually by their peers and the people whom they supervised, materials for engagement were evaluated on a monthly basis by all members of the UCMI team and our STP collaborators, CHAs evaluated the communication skills of their peers in group practice and trainings, and regular social media and national media outlets shared contact information that people could use to communicate with the engagement team about questions, concerns, or doubts they had related to the UCMI program.

Information obtained through evaluations, assessments, surveys, and direct contact connected engagement program decision-making back to the end-user group that was most directly affected.

## RESULTS

Initial and regular contact and information sharing with STP stakeholders was important in the establishment of transparency and trust. The first 2 years of robust stakeholder engagement resulted in UCMI understanding of the STP health system, governance, historical malaria control, and engagement efforts and in stakeholder understanding of the UCMI program objectives, goals, and gene-drive technology. It resulted in the establishment of an engagement team structure that respected existing roles and functions and one that remains stable regardless of national politics and leadership changes.

Stakeholder engagement has helped to build a general understanding that the program is collaborative and keeps information available and accessible. Early stakeholder engagement was critical in establishing program credibility regarding our commitment to a model that shifts decision-making to end-user groups and later in continued acceptance of the operational and administrative structure of the UCMI program in the STP. This is especially important in a dynamic political environment where stakeholders in decision-making positions regularly change, but the foundational structures and systems remain the same. The challenge in applying the RBM in a dynamic environment is the continuous need to begin at the beginning with new stakeholders and new collaborators who were not involved in earlier elements of program development and who often have different priorities, beliefs, and opinions about how the work should be done. Robust stakeholder engagement efforts that expand beyond current government leadership to all members of civil society is one way that the UCMI program is attempting to address this challenge, building a channel of communication and information sharing between groups of people that inform political leaders and those who may be leaders themselves in the future.

The initial community assessment provided information identifying community leaders and health professionals in their communities as preferred sources for health information. Early establishment of a stakeholder engagement group, leadership team (health delegates, malaria focal points, and manager), and integration of these information sources in the engagement program has provided credibility and added value to all engagement decisions that have been made. When questions or concerns arise, community health leaders are the first point of contact for more information. Despite how each individual community member may feel about the UCMI-modified mosquito, there is trust in the information shared with them about the technology because the public health professionals and community leaders they trust have helped develop, design, and deliver it. Materials were developed in the local language and with visuals that were socially acceptable. The engagement plans and engagement program structure were developed and supported by individuals and the organizations that are directly involved in the management and planning of the engagement work and by those who hold understanding of the national history, beliefs, and health priorities. This created conditions in which decisions made about the who, how, where, and when of program engagement activities were made by those who were in the best position to determine the adequacy and efficacy of these decisions. It provided an environment that allowed the program to conduct a nationwide community assessment within 2 months of the establishment of the local team. This level of engagement with community members was immediately made possible because the team was local and trusted. The size, scope, and expertise of the local team provided the program with an immediate connection to people throughout the country, of all ages and backgrounds. This facilitated community input into the earliest phases of engagement planning and assurance that the program was reaching as many people as possible.

Feedback from the CHAs and community members during this first phase of engagement led to the development of engagement materials (printed educational booklets for children in the schools, animated videos, and posters) to reinforce specific concepts and promote understanding and awareness. Early community feedback reinforced a need to establish a strong presence on social media and on national TV and radio. In 2022 and 2023, the team developed 12 unique animated videos and corresponding audio spots for media. The team has also established regular radio shows on local radio stations in Príncipe and the district of Lobata, where the engagement teams invite community member participation to share views and opinions about the program and to ask questions and answer questions related to the program. Most recently, in response to stakeholder feedback, the team conducted open public debate and discussions related to the program. These methods encouraging open dialogue and discussion provide opportunities for managing expectations and increasing awareness about project work, potential implementation, timelines, and government decision-making and regulation surrounding the technology.

The use of visual materials was an important way that people wanted to receive information. This moved the program to partner with a local graphic designer and biology teacher by training who worked with the engagement team to develop digital and printed content to complement awareness activities. The designer also developed training materials for use with team trainings to help simplify and clarify information. The UCMI digital and printed engagement materials are regularly shared on the program’s Facebook page @UCMISTP. Materials are also made available on the UCMI website (www.stopmalaria.org) and on the project YouTube channel @ucmi-iniciativacontraopalu4409.

After the engagement team had been working in the communities for the first full year, an exam was conducted for all members of the team to assess knowledge and skills. The exam was a compilation of questions that had previously been provided on pre- and posttests from the trainings. Based on the results of the evaluation and feedback from the team and communities, the team composition was adjusted to include additional teachers for each district team to replace those agents who were underperforming. The CHAs reported in monthly team meetings, feeling an increased level of support when teachers were present during engagement activities. They expressed that teachers were able to easily answer scientific questions and had helpful communication skills that could be applied while engaging with community members. Increasing the number of teachers within each district team increased this level of support and allowed the team to have greater reach in schools across all districts. Teacher engagement in the schools has become an integral part of the program and a much-requested service by school administrators, students, and parents. The team has developed a children’s malaria activity book for the program that is now being integrated into the curriculum in primary schools in São Tomé. Completion, review, and evaluation of the book involves not only the students, but parents and schoolteachers as well. Requests for this level of engagement in schools continue to increase, and the engagement team has developed a full engagement plan for activities in schools across the country in the next year. These activities take the same phased approach as those in the communities, integrating information about malaria biology and transmission into the existing curriculum and activities that build on scientific concepts related to the UCMI technology, leading to discussions and activities about how the UCMI-modified mosquito works and its potential use for malaria control. Engagement in schools gives the engagement team opportunities to interact directly with youth in the STP and respond to their specific questions, perceptions, and concerns related to malaria and the modified mosquito. The engagement in schools has also integrated school administrators and teachers into the planning of activities specific for their school. The RBM calls for this level of flexibility and collaboration so that with each phase of research, engagement teams and activities can adapt and expand to the needs and concerns of each group. Over the last 2 years, we have received many positive comments from the public (E-mails, phone calls, and on Facebook) about our program commitment to engagement and the inclusion of all groups beyond stakeholders and government officials. This was possible very early because of the establishment of strong local teams.

National community assessments have allowed the team to integrate voices of community members into the development process. The assessment results provided valuable information to the team regarding community understanding and retention of information shared, knowledge gaps, and program perceptions and assumptions. After the second assessment was completed in November 2022, the team came together for a 3-day training and evaluation of results and assessed and discussed differences between communities and districts and areas where improved engagement efforts were needed. The assessment gave the team important community feedback about the information that was being shared and how it was shared. Through the assessment, the voices and perceptions of community members were integrated into the development process of future engagement activities. Importantly, the assessment identified specific assumptions, concerns, and questions about the program. It clearly showed gaps where information sharing had been missed or misunderstood. In many cases, these misconceptions came from districts where the engagement team and activities were not as strong as in others, and several communities in the district had been missed or not engaged as often. Six specific community concerns and questions were identified, and the team worked with the graphic designer to create 60-second animated explanatory videos to address these concerns (Table 3). For example, one concern was that the program had previously released a modified mosquito into the natural environment in the STP. The video addressed this using animated characters and simple, conversational dialogue to explain that the program has not released a modified mosquito, why this is not possible now, and what needs to be done for the appropriate authorities to make that decision. These short videos were shown on national TV, radio, and social media for several months. Community inclusion and active participation in the development of engagement activities helped the team to ensure shared understanding and program transparency, and it strengthened the development of tools and resources used to share important program information. Perhaps more importantly, it assisted in the continuous improvement and evaluation of the program’s work and held the entire team accountable to the RBM.

Another important result of the second community assessment was the identification of the need for increased support and capacity sharing on the island of Príncipe. Príncipe, although just over 150 km from the larger island of São Tomé, is relatively isolated from the day-to-day work of the larger program team. It was clear from assessment results that community members in Príncipe had little to no understanding of the UCMI program and had some important knowledge gaps related to malaria in general. It was a challenge for the engagement manager to provide regular and consistent support to the Príncipe engagement team from a distance. Although the engagement team structure was the same, the health delegacy, malaria control team (and focal point), and general health system had far fewer human and financial resources than those on the larger island, and the responsibilities of these roles were often far greater than they were in São Tomé. The manager suggested that a full-time engagement focal point in Príncipe be considered and a scope of work and a recruitment and training plan developed. One of the important requirements for focal point candidates was that the individual must be a resident in Príncipe and have knowledge and understanding of the local communities, governance, and civil society. A full-time focal point was hired in March 2023, and within 3 months the team received two refresher trainings and was actively engaging with communities and stakeholders on the island. The team will be conducting a follow-up assessment in early 2024, and there is an expectation that results will show improved awareness and understanding given the increased unity and strength of the team.

In addition to assessments conducted by the engagement team, there has been discussion about the value of assessing community perceptions of the program by individuals who are not directly involved in the program. The UCMI will be conducting an external and independent assessment of the engagement program and an evaluation of community perceptions and acceptance of the proposed technology in the last quarter of 2024.

## DISCUSSION

The RBM provides a framework that shifts the decision-making surrounding the development of an engagement program, strategies, and activities to end-user groups. It is a model that provides an opportunity for stakeholders and community members to actively participate and contribute to the decision-making process surrounding program engagement. We demonstrated here how the RBM was applied in the development of a national engagement program for gene-drive research and how it influenced the way that decisions were made. Information and feedback that the UCMI team has received from community assessments, workshops, stakeholder meetings, educational programs, and regular community engagement activities play a huge role in the ongoing and regular development and refinement of program communication methods to support the model. How people share information, where and how they feel comfortable doing it, and when, who, and how to engage varies from place to place, community to community, and country to country. This model provides a framework for identifying end-user preferences, existing knowledge, resources, and relationships and a method for integrating this information directly into the decisions involved in developing an engagement program, strategy, and activities.

The RBM is a model that has been tried and tested with success in other public health interventions and applications, and here we demonstrated how the model was scaled up and applied to a national engagement program for gene-drive research. The RBM is not a one-size-fits-all approach; it provides a framework that supports integration of end users in the development of the engagement program from start to finish, and this will look very different from one program to the next. Gene-drive research is preparing to advance into the next phase, testing the technology via small, confined field trials.^3^ The UCMI has applied the RBM for engagement as it prepares to enter this phase of research, offering an opportunity to assess the application of this engagement model prior to applying it at a larger scale should the technology advance beyond the current phase. We present here the application of the RBM in the STP and share how the model facilitated advancement of engagement across the entire country while integrating stakeholder and community input into all planning stages of the program. Although the STP is a small country, where diversity (ethnic, linguistic, etc.) is lower than in other, much larger, more populated countries where the technology may eventually be deployed, we believe that the same approach can be applied with a scaling up of resources and time, particularly in the early stages of initial stakeholder engagement and shared goal setting.

The implementation of the RBM in the STP was not without challenges, some of which were previously mentioned in the Results section of this paper. Within the context of the UCMI program, the primary strength of this model is also its primary challenge, shifting decision-making to those entirely outside of the research program. It is easy, after implementation, to focus on the benefit of creating an engagement program designed by the very same individuals it was designed to engage and who are active members of the end-user communities. This can be very difficult for the development team who not only has a program goal, timeline, and funder expectations to meet but who also has preconceived ideas about how the work should be done. Early application of the RBM required regular and rigorous program team discussions about the commitment to the model. Often questions arose regarding content development, methods for communication, etc., that would require a pause and reorientation of the model with the question “What end-user groups need to be involved in this decision?” and then shifting this question back to our stakeholders. This is very difficult to apply in daily practice. It can slow the pace at which the program advances, and it requires additional time for building the capacity and knowledge of the program team about local understanding, perceptions/awareness, and expectations. This is an important consideration when considering the application of the model for larger scale deployment. Engagement efforts across large, diverse countries that integrate existing knowledge and perspectives will require additional time, early planning, and resources.

Early application of existing protocols or best practices that seemed easily transferable and translatable without active engagement and involvement of end-user groups provided useful learning opportunities for the program team. It was critically important to apply existing end-user practices, protocols, knowledge, and perspectives into decisions surrounding all program practices. A benefit of the model is that it requires developers/practitioners to meet end-user groups where they are in the context of their experiences, knowledge, and perspectives. By doing this, the stakeholders and community groups in the STP had a much deeper understanding of program work and took ownership and responsibility for the engagement practices developed for the program. Relationships and shared understanding as a result of the model offer a mechanism for managing challenges and differing opinions related to practices and protocols. Early development of shared goals and objectives and regular dialogue provide an environment where collaborative agreement on important decisions can more easily be achieved.

Shifting management to an entirely local, end user–based team was not intuitive or entirely comfortable. Stakeholders expressed doubts about local capacity, knowledge, and skills required for the work; however, we found talent, skill, and expertise that was discovered to be extraordinary. Despite the size, economic status, and limited access to education in the STP, there is a deep pool of talent and interests. The RBM model begged us to set aside assumptions, and perhaps previous experience, and build on the strengths and contexts of the specific site/community where the work was being conducted.

Finally, conducting engagement, regardless of the model or framework, involves dynamic human populations that are continuously responding to complex and dynamic social, economic, and political challenges. The model attempts to address this through involvement of the population in the development of engagement activities and communication strategies that are most acceptable for them; however, day-to-day challenges of basic living take priority, and when these challenges are many, engagement can be difficult. This challenge was further complicated when engaging individuals about a complex novel technology that is designed to address a specific public health concern but does not offer solutions to the many other concerns of equal or greater importance they may be facing in the moment.

Fatigue was a regular topic of conversation within the engagement team, and they took this into consideration at each phase of engagement. The team is currently developing new communication and information-sharing tools that integrate engagement with community activities. Examples include community cleanup days that focused on removal of litter and garbage that provided mosquito breeding habitats and community games, trivia, and tournaments. This challenge required creative planning and the ability to respond to feedback from communities.

Although the model cannot resolve all the engagement challenges and questions surrounding the application of gene-drive technology, the RBM does provide a tested approach for successful end-user development of effective engagement strategies through establishment of relationships and shared understanding. An exact replication of the UCMI application of the model in the STP would contradict the model itself. What can be replicated are the strategies used to apply and interpret the concepts of the model. The aim of this paper was to demonstrate how the UCMI translated the model from paper to practice with practical examples and effects in the hope that it would be a useful reference and tool for projects looking to establish a model for engagement that involves direct participation of end-user groups.

## Supplemental Materials

10.4269/ajtmh.23-0862 Supplemental Materials
