# Supplementary material for: Practical Application of a Relationship-Based Model to Engagement for Gene-Drive Vector Control Programs
Source: Am J Trop Med Hyg. 2024 Jun 18;111(2):341–60. doi: 10.4269/ajtmh.23-0862 (PMC11310621; doi:10.4269/ajtmh.23-0862)
Supplement: Supplemental Materials [file tpmd230862.SD1.pdf]

Supplementary Table I.
Community Assessment Data for Assessments I and II

2021 – Community Assessment I

Total Respondents: 557 **Multiple Responses: *** **Free Response: ****

Question 1. Which district is this assessment from?

Answer Choices	Respondents
Água Grande	144
Lobata	80
Lembá	56
Cantagalo	37
Caué	40
Mé-Zóchi	88
Príncipe	106
No response	6

Question 2. Which of the following options best describe how you feel about malaria?

Answer Choices	Respondents
I'm not worried	97
I'm worried	434
I don't know	19
No response	7

Question 3. Doo you feel presence of malaria in your country has (select best response):

Answer Choices	Respondents
Worsened	95
Remained the same	85
Improved	371
No response	6

Question 4. What do you know about how humans are infected with malaria?

Answer Choices	Respondents
Nothing	62
Little	392
A lot	99
No response	4

Question 5. How safe do you consider aerosols with insecticides (insecticide sprays, IRS) for the control of malaria?

Answer Choices	Respondents
Very safe	121
Safe	296
I don’t know	61
They are not safe	73
No response	6

Question 6. How effective do you consider aerosols with insecticides (residual indoor spraying, IRS) for the control of malaria?

Answer Choices	Respondents
Very effective	138
Effective	310
I don’t know	46
They are not effective	56
No response	7

Question 7. How safe do you consider insecticide-treated mosquito nets (long-lasting insecticidal net, LLIN) for the control of malaria?

Answer Choices	Respondents
Very safe	205
Safe	321
I don’t know	17
Are not safe	5
No response	9

Question 8. How effective do you consider insecticide-treated mosquito nets (long-lasting insecticidal net, LLIN) for the control of malaria?

Answer Choices	Respondents
Very effective	193
Effective	327
I don’t know	22
They are not effective	4
No response	11

Question 9. How safe do you consider antimalarial medications for the control of malaria?

Answer Choices	Respondents
Very safe	185
Safe	301
I don’t know	44
They are not safe	6
No response	21

Question 10. How effective do you consider antimalarial medications for the control of malaria?

Answer Choices	Respondents
Very effective	144
Effective	345
I don’t know	43
They are not effective	4
No response	21

Question 11. What are your current sources of information about malaria? *

Answer Choices	Respondents
Community meetings	238
Clinics	52
Community health professionals	209
Community leaders	108
PNLP	84
Television/Radio	367
Printed materials	18
Others	3
No response	16

Question 12. How satisfied are you with the information you get about malaria?

Answer Choices	Respondents
I'm not satisfied	60
I don’t know	30
I'm satisfied	441
Skipped	26

Question 13. How would you prefer to obtain information about malaria? *

Answer Choices	Respondents
Community meetings	320
Clinics	56
Community leaders	214
Television/Radio	337
Printed materials	42
Others	0
No response	19

2022 – Community Assessment II

Total Respondents: 1973 **Multiple Responses: *** **Free Response: ****

Question 1. Which district is this evaluation from?

Answer Choices	Respondents
Água Grande	421
Mé-Zóchi	326
Príncipe	300
Lembá	242
Cantagalo	210
Caué	180
Lobata	294

Question 2. Gender of respondent

Answer Choices	Respondents
Masculine	863
Feminine	941
No response	169

Question 3. In your opinion, are malaria cases increasing in São Tomé?

Answer Choices	Respondents
Yes	288
Remained the same	333
Decreased	1342
No response	10

Question 4. How do you feel about the malaria control methods used in São Tomé and Príncipe?

Answer Choices	Respondents
Very effective	778
Effective	851
Not very effective	323
No response	21

Question 5. In your opinion, how is malaria transmitted? **

Open Answer Responses	Respondents
Bites from infected mosquitoes	803
Bites from mosquitoes	1049
Stagnant water, garbage, dirty things	150
From a person with malaria	13
Other	12

Question 6. Does the mosquito that transmits malaria also transmit dengue?

Answer Choices	Respondents
Yes	42
No	1666
I don’t know	255
No response	10

Question 7. What sources of information do you usually use to learn about malaria in São Tomé and Príncipe? *

Answer Choices	Respondents
Community meetings	932
Health centers	905
Malaria control program	845
Awareness activities	1223
Health professionals in the community	893
Radio	1244
Television	1276
Printed materials	253
Social media	76
Other	122

Question 8. Do you have any knowledge about the UCMI project?

Answer Choices	Respondents
Yes	1444
No	508
No response	21

Question 9. What are your current sources of information about the UCMI project? *

Answer Choices	Respondents
Community meetings	706
Awareness activities	1111
Healthcare professionals in the community	743
Radio	655
Television	854
Facebook	139
Printed material	197
Other	12

Question 10. How do you prefer to obtain information about the UCMI project? *

Answer Choices	Respondents
Community meetings	1056
Awareness activities	1420
Healthcare professionals in the community	1082
Radio	1168
Television	1243
Facebook	504
Printed material	372
Other	86

Question 11. Do you follow the UCMI project posts on Facebook?

Answer Choices	Respondents
Yes	267
No	1636
No response	70

Question 12. Does the UCMI project want to eradicate mosquitoes in São Tome and Príncipe?

Answer Choices	Respondents
Agree	367
Disagree	1336
I don’t know	246
No response	24

Question 13. Does the UCMI project want to eliminate malaria with modified mosquitoes?

Answer Choices	Respondents
Agree	1711
Disagree	69
I don’t know	157
No response	36

Question 14. What activities is the UCMI currently developing in São Tomé and Príncipe? **

Answer Choices	Respondents
Mosquito collection and analysis	743
Technician training and capacity building	610
Community awareness	875
All of these	772
I don't know	251

Question 15. In your opinion, what is the goal of the UCMI project? **

Answer Choices	Respondents
I don’t know	290
Eradicate mosquitoes	60
Eradicate malaria	1336
Eradicate malaria through modified mosquitoes	187
Other	52
No response	48

Question 16. In your opinion, will the UCMI project contribute to the elimination of malaria in São Tomé and Príncipe?

Answer Choices	Respondents
Yes, a lot	1514
A little	115
Maybe	276
It won't change anything	23
No response	45

Question 17. Have you heard about genetically modified mosquitoes?

Answer Choices	Respondents
Yes	1388
No	518
No response	67

Question 18. In your opinion, are there genetically modified mosquitoes in São Tomé and Príncipe?

Answer Choices	Respondents
Yes	80
No	1417
I don't know	423
No response	53

Question 19. Have you heard about the mark-release-recapture study of mosquitoes that the UCMI project has been conducting?

Answer Choices	Respondents
Yes	1427
No	492
No response	54

Question 20. In your opinion, have the mosquitoes used in this mark-release-recapture (MRR) study been modified?

Answer Choices	Respondents
Yes	126
No	1375
I don’t know	428
No response	44

Question 21. Do you believe that the UCMI project should continue its awareness and community engagement activities in São Tome and Príncipe?

Answer Choices	Respondents
Yes	1876
No	40
No response	57
